# Integration of External Vagus Nerve Stimulation in the Physiotherapeutic Management of Chronic Cervicogenic Headache: A Case Report

**DOI:** 10.3390/healthcare13162030

**Published:** 2025-08-17

**Authors:** Rob Sillevis, Nicola Khalaf, Valerie Weiss, Eleuterio A. Sanchez Romero

**Affiliations:** 1 Department of Rehabilitation Sciences, Florida Gulf Coast University, Fort Myers, FL 33965, USA; nvarveris@fgcu.edu (N.K.); vweiss@fgcu.edu (V.W.); esanchezromero@fgcu.edu (E.A.S.R.); 2 Research Group in Nursing and Health Care, Puerta de Hierro Health Research Institute-Segovia de Arana (IDIPHISA), 28222 Majadahonda, Spain; 3 Interdisciplinary Research Group on Musculoskeletal Disorders, 28014 Madrid, Spain; 4 Physiotherapy and Orofacial Pain Working Group, Sociedad Española de Disfunción Craneomandibular y Dolor Orofacial (SEDCYDO), 28009 Madrid, Spain

**Keywords:** cervicogenic headache, manual therapy, vagus nerve stimulation, physical therapy modalities, neuromodulation, atlanto-axial joint

## Abstract

**Background:** Cervicogenic headache (CGH) is a prevalent secondary headache disorder associated with upper cervical spine dysfunction, often involving nociceptive convergence at the trigeminocervical complex. While manual therapy and exercise have demonstrated benefit, autonomic dysregulation may contribute to persistent symptoms. This case report explores the integration of external vagus nerve stimulation (eVNS) into a multimodal physical therapy approach targeting both mechanical and neurophysiological contributors to CGH. **Case Description:** A 63-year-old female presented with chronic CGH characterized by right-sided suboccipital and supraorbital pain, impaired sleep, and postural dysfunction. Examination revealed a right rotational atlas positional fault, restricted left atlantoaxial (AA) mobility, suboccipital hypertonicity, and reduced deep neck flexor endurance. Initial treatment emphasized manual therapy to restore AA mobility and atlas symmetry, combined with postural correction and neuromuscular training. **Intervention:** After initial symptom improvement plateaued, eVNS targeting the auricular branch of the vagus nerve was introduced to modulate autonomic tone. The patient used a handheld eVNS device nightly over three weeks. **Outcomes:** Substantial improvements were observed in the Neck Disability Index (↓77%), Headache Disability Inventory (↓72%), and pain scores (↓100%). Cervical mobility, atlas symmetry, and deep neck flexor endurance improved markedly. The patient reported reduced anxiety, improved sleep, and sustained headache relief at one-month follow-up. **Conclusions:** This case highlights the potential synergistic benefits of integrating eVNS within a physiotherapy-led CGH management plan. Further research is warranted to explore its role in targeting autonomic imbalance and enhancing conservative treatment outcomes.

## 1. Introduction

Headaches are a major global health concern, contributing substantially to disability, reduced productivity, and diminished quality of life [1]. Among the various headache types, cervicogenic headache (CGH) emerged as a distinct clinical entity in the 1980s, when the cervical spine was first implicated as a causative factor in a subset of headache disorders [2]. The International Headache Society (IHS) defines CGH as “*a headache caused by a disorder of the cervical spine and its component bony, disc, and/or soft tissue elements, usually but not invariably accompanied by neck pain*” [3].

The true incidence of CGH remains unclear, with reported prevalence ranging from 0.17% to 64.5%, a disparity likely attributable to differences in study populations, diagnostic criteria, and clinical settings [4]. Despite this variability, CGH is estimated to account for up to 20% of all chronic headache presentations [3], posing a frequent diagnostic and therapeutic challenge in both primary care and specialized clinical environments [2,4,5]. While its exact pathophysiology is not fully understood, current evidence highlights a central role for upper cervical dysfunction, particularly at the C1–C3 levels [6,7].

Anatomical studies have shown direct connections between the trigeminal nerve and the upper cervical spinal nerves through the trigeminocervical nucleus [8,9]. This anatomical convergence allows nociceptive input from the cervical region to refer to pain to the forehead and facial structures [10]. Additionally, aberrant upper cervical kinematics in combination with muscular dysfunction have also been implicated in CGH [11]. The suboccipital region contains myodural bridges between the obliquus capitis inferior, rectus capitis posterior minor, and rectus capitis posterior major muscles and the dura mater [12,13]. Recently, direct collagenous attachments linking the outer layer of the dura mater to the nuchal ligament have been identified, suggesting a structural and potentially functional continuum between these tissues [14]. These fascial–dural attachments suggest that cervical movement and muscular tension may influence the dural mechanics and contribute to nociceptive sensitization. This is consistent with findings of positive neural tension tests in CGH patients [15,16,17]. Suboccipital muscle activity, particularly contraction of the obliquus capitis inferior muscles, will result in ipsilateral atlas rotation relative to the axis, potentially increasing the dural tension [18,19]. Positional asymmetry of the atlas has been linked to headache intensity, reinforcing the importance of unimpaired upper cervical mobility in CGH [18,19,20]. While these mechanical and neuroanatomical mechanisms are central to CGH, emerging evidence indicates that they may also interact with dysregulation of central pain-modulatory systems, including the autonomic nervous system [21,22,23]. This link provides a physiological bridge between cervical musculoskeletal dysfunction and the systemic pain and sensory alterations observed in CGH.

Although mechanical dysfunction is a key factor, CGH also involves neurophysiological and psychosocial dimensions [21]. Dysregulation of the autonomic nervous system (ANS) is frequently observed in patients with pain and conditions such as migraine headaches and cervical myofascial pain syndrome [22]. Heightened sympathetic activity can trigger vasoconstriction, altered nociception, and impaired homeostasis, thereby intensifying painful conditions [22,23]. Conversely, parasympathetic activity, which is mediated in part by the vagus nerve, can dampen pain perception, reduce inflammation, and promote homeostatic recovery [24]. Prolonged sympathetic dominance will result in vagal inhibition. Reduced vagal tone has been associated with increased muscle tone in the head, neck, and shoulders, possibly contributing to cervical dysfunction and CGHs [24]. These contrasting roles of the SNS and vagus nerve suggest that targeting autonomic regulation may be a promising approach in the management of CGH.

**Figure 1:**
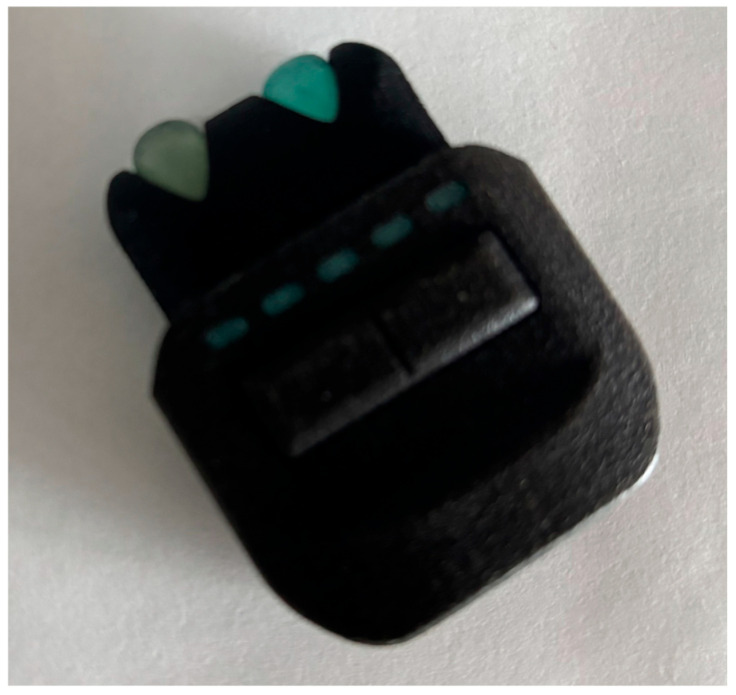
eVNS.

Interventions aimed at restoring autonomic balance, such as vagus nerve stimulation (VNS), may reduce heightened sympathetic activity and subsequently decrease pro-inflammatory and vasoactive responses that contribute to the sensitization of trigeminovascular pathways [24,25]. VNS modulates afferent input from the cervical vagus nerve and has been explored as a neuromodulatory treatment for pain [26]. Although traditional VNS involves surgically implanted electrodes, recent advances have enabled noninvasive approaches. External Vagus Nerve Stimulation (eVNS) specifically targets the auricular branch of the vagus (AbV) and represents a promising alternative [27,28] (Figure 1).

Although the precise mechanism is unclear, both neurophysiological and neuromodulatory effects might contribute to the positive effects of eVNS. Emerging evidence suggests that VNS may reduce migraine headache frequency and intensity by influencing central pain processing pathways and attenuating nociceptive signaling associated with cervical spine dysfunction [29]. The AbV projects afferent fibers to the nucleus tractus solitarius within the brainstem, facilitating downstream modulation of higher autonomic control centers, including the amygdala [30,31]. A recent meta-analysis found moderate to high-quality evidence suggesting that eVNS is well tolerated and beneficial in reducing headache frequency [32]. Direct AbV stimulation with eVNS impacts the neurotransmitter system, including the release of norepinephrine and serotonin, and thus influences mood and pain modulation [33]. In patients with migraine, daily use of eVNS has demonstrated comparable efficacy to morphine [34]. By enhancing descending inhibitory control, eVNS may offer a safe opioid-sparing strategy for trigeminal pain syndromes.

Given its ability to regulate autonomic function and modulate central pain pathways, eVNS represents a promising adjunct to the multimodal management of CGH [35]. The integration of external vagus nerve stimulation (eVNS) with established pharmacological treatments and non-pharmacological approaches, including manual therapy, therapeutic exercise, and patient education, may offer a more comprehensive management strategy for CGH [36]. This multimodal approach targets both peripheral dysfunction and central sensitization mechanisms underlying CGH pathophysiology. Emerging evidence suggests meaningful clinical potential of eVNS for patients with CGH [37].

However, key clinical parameters, including the ideal stimulation site, frequency, and dosage, remain undefined. Therefore, further studies are warranted. The presentation of this case report aimed to illustrate the clinical application and potential benefit of integrating eVNS within a physiotherapy-led intervention for a patient with chronic CGH.

## 2. Materials and Methods

### 2.1. Case Report: Patient History

A 63-year-old mesomorphic female presented with direct access to physical therapy for the evaluation and management of worsening headaches. Her neurologist had recently diagnosed her with CGH. Her chief complaints included frequent headaches and neck pain that gradually intensified over the previous months, limiting her sleep quality and daily functioning. She reported pain onset in the occipital region radiating to the supraorbital area, predominantly on the right side. The neck pain and headaches were aggravated by cervical rotation (especially to the left), extension, and overhead tasks. Based on her presentation, aggravating factors, and previous positive response to nerve blocks, she met the International Classification of Headache Disorder 3 Criteria 11.2.1 for CGH [38]. Her symptoms were associated with anticipatory anxiety and behavioral avoidance due to the fear of triggering a headache episode.

She reported a history of vascular migraines, diagnosed at age 18, with intermittent episodes persisting into adulthood. Previous interventions included chiropractic manipulation (most recently six years prior to the current presentation), multiple courses of physical therapy incorporating joint manipulation, modalities, and exercise, as well as various pharmacological treatments. At the time of presentation, she continued to use naproxen and Imitrex. Additional past treatments included facet joint injections (C5–C7 bilaterally) and suboccipital nerve blocks, with the most recent injections administered three years earlier. These strategies have provided partial and inconsistent relief. Imaging of the cervical spine and brain revealed only age-appropriate findings, including disc degeneration and uncovertebral arthrosis at C5–C7.

The patient had previously received care from the treating clinician (R.S.) during two separate episodes within the last four years. During those prior treatment periods, she experienced a notable reduction in headache frequency, reporting a decrease to 2–4 headache days per month. Interventions during those episodes focused on manual therapy techniques targeting the atlantoaxial joint, suboccipital active mobility, and neuromuscular control. Additionally, she was prescribed a home exercise program that emphasized self-mobilization techniques, deep cervical flexor strengthening, and postural awareness. These interventions collectively contributed to meaningful symptom relief at the time.

At the time of her evaluation, the patient reported a recent increase in headache frequency to 2–3 days per week, each significantly impairing her functional activities. Additionally, she reported difficulties sleeping due to neck pain and just being uncomfortable in bed. She described anticipatory anxiety and behavioral avoidance associated with fear of headache onset, suggestive of affective components influencing her condition. Pain intensity ranged from 3/10 to 9/10 on the Numeric Pain Rating Scale (NPRS); at the time of the assessment, this was rated 7/10. Headaches typically began in the occipital region and progressed to supraorbital pain, predominantly on the right and typically would last all day. Accompanying neck pain was provoked by cervical rotational motion, especially left, cervical extension, and overhead tasks. She denied symptoms of dizziness, sensory changes, balance impairment, or alterations in vision, hearing, or olfaction. Her general medical history was noncontributory, and a general health screening was negative for red or yellow flags, supporting the appropriateness of proceeding with further musculoskeletal evaluation and treatment.

To objectively assess her condition, the patient completed validated self-report measures. Neck pain intensity was measured using the Visual Analog Scale (VAS), which has demonstrated strong validity and reliability for assessing both acute and chronic pain conditions [39,40]. The patient reported her pain intensity as 0.0 mm at its best, 90.0 mm at its worst, and 42 mm at the time of the evaluation, indicating a moderate level of current discomfort. To further evaluate disability related to neck pain and headaches, the patient completed the Neck Disability Index (NDI) and the Headache Disability Inventory (HDI). The NDI is a widely used 10-item questionnaire designed to assess perceived neck-related disability, with higher scores indicating greater functional impairment [41,42,43,44,45]. At the time of assessment, the patient’s score was 58 out of 100, suggesting a moderate level of disability. The NDI has demonstrated strong content and construct validity as well as test-retest reliability in populations with neck pain [41,46].

The HDI, a 25-item measure assessing the impact of headaches on daily functioning, includes two subscales: 12 functional and 13 emotional items, with a total possible score of 100. Higher scores indicate a greater perceived disability. The patient’s score was 72 out of 100, reflecting the severe impact of headaches on her daily life. The HDI has also demonstrated sound construct validity and test-retest reliability, supporting its clinical utility in headache populations [47].

### 2.2. Examination

The patient presented with a normal gait and appeared comfortable during the clinical interview. Postural assessment revealed a forward head posture with compensatory suboccipital extension, bilateral shoulder protraction (more pronounced on the right), and scapular winging on the right side. Forward head posture has been associated with various musculoskeletal impairments, including neck pain, headache, and craniofacial dysfunction [48,49,50,51,52,53].

Cervical active range of motion (AROM) was assessed in a seated position using an inclinometer for flexion, extension, and lateral flexion, and a goniometer for rotation. AROM assessment is considered a valuable component of clinical evaluation in patients presenting with cervicogenic headaches (Table 1) [54].

The patient exhibited reduced cervical rotation to the left compared to the right. Forward flexion and lateral flexion bilaterally provoked a sensation of “muscle tightness,” while extension and left cervical rotation reproduced her characteristic neck pain.

Further provocation testing revealed pain during the right-sided Spurling’s test and the right Quadrant test, though no radicular symptoms were elicited. These findings suggest the involvement of mid-to-lower cervical facet joints, which may contribute to the patient’s observed limitations in AROM [55]. Neurological screening results were unremarkable: cranial nerve function was intact, upper limb myotomes were symmetrical, reflexes were normal, and upper limb tension tests were negative.

Palpation revealed elevated muscle tone in the suboccipital triangle, scalene, levator scapulae, and upper trapezius muscles, with greater tonicity noted on the right side. Positional palpation of the upper cervical spine identified an asymmetry of the atlas in the transverse plane. Specifically, the left transverse process appeared to be positioned more anteriorly relative to the right when palpated with the patient seated and maintaining mandibular protrusion. This transverse plane asymmetry of the atlas has previously been identified as a common rotational positional fault and has been associated with cervicogenic headaches [18,56,57].

Upper cervical ligament integrity was assessed with the seated Sharp-Purser and supine alar ligament stress tests, both of which were negative, ruling out ligamentous instability [58]. Next, the presence of upper cervical dysfunction was assessed using passive intersegmental mobility testing. The AA joint was specifically examined using the cervical flexion-rotation test (FRT), performed with the patient in a supine position. During the FRT, the cervical spine was placed in maximal flexion to isolate motion at the AA joint, followed by a passive rotation of the head to both sides. The range of motion was measured using a goniometer, and the quality of the end feel was determined [59]. The FRT has demonstrated strong reliability and diagnostic utility for patients with cervicogenic headaches. Hall et al. [60] reported high sensitivity (90–91%), specificity (88–90%), and an overall diagnostic accuracy of 91%, supporting its clinical value [60,61]. In this case, the FRT revealed decreased left rotation, identifying a hypomobility on the right. Testing caused symptom reproduction, indicating that dysfunction of the right AA joint was likely a significant contributor to the patient’s headache presentation.

To assess additional cervical segments, spinal segmental motion palpation was performed. While the validity and reliability of spinal motion assessment vary from poor to good depending on the technique and examiner training, it remains a frequently used component of clinical reasoning in manual therapy [62,63,64,65]. A down-slide test was used to assess the mid-to-lower cervical facet joints (C2–C7), and hypomobility was noted on the right at the C5–C7 levels [66], was used to assess the mid-to-lower cervical facet joints (C2–C7), and hypomobility was noted on the right at the C5–C7 levels. Thoracic spine mobility testing revealed restricted extension mobility at T2–T5, which may also have contributed to altered regional mechanics and symptom persistence.

The patient’s postural presentation of forward head posture is consistent with the muscle imbalance patterns described in upper crossed syndrome, which often results in adaptive shortening and weakness of upper quadrant musculature [67]. Manual muscle testing revealed weakness of the short neck flexors, which were graded as 4/5 [68]. Endurance of the deep cervical flexors was evaluated using the method described by Jarman et al. [69], who demonstrated moderate to good interrater reliability. According to normative data, healthy females should be able to maintain the test position for 30 s. In this case, the patient was able to sustain the position for only 14 s, indicating reduced muscular endurance, and was rated as fair.

Additional muscle testing showed bilateral weakness of the rhomboids and middle trapezius, both graded at 4/5. Muscle length assessment revealed shortening in the suboccipital muscles, levator scapulae, upper trapezius, and pectoralis muscle groups, with greater involvement on the right side. These findings further support a pattern of muscular imbalance, possibly contributing to her postural dysfunction and symptom presentation.

### 2.3. Clinical Impression

The clinical impression was formulated based on a comprehensive physical therapy evaluation of the cervical spine, incorporating both mobility and motor control assessments. It was hypothesized that the patient exhibited a right rotational positional fault of the atlas, accompanied by hypomobility of the right AA joint and right-sided facet restrictions at the C5–C6 level. Both provocation testing and palpatory asymmetries supported these findings.

Additionally, the patient demonstrated impaired postural awareness and notable muscle dysfunction throughout the upper quarter, including weakness and endurance deficits of the deep cervical flexors and scapular stabilizers, as well as soft tissue shortening in several key muscle groups. These mechanical impairments were believed to contribute to the patient’s cervicogenic headache symptoms and persistent neck pain. Based on her response to prior care and current presentation, the prognosis for functional improvement with targeted intervention was considered good.

### 2.4. Plan of Care

Following the initial evaluation, the patient attended one session per week for four consecutive weeks. Visit 6 occurred two weeks after Visit 5, and the final treatment session took place four weeks after Visit 6. The total duration of care was 10 weeks. At each visit, the patient was re-evaluated, and interventions were tailored to the mechanical dysfunctions identified during that session. Outcome measures—including the Neck Disability Index (NDI), Headache Disability Inventory (HDI), Numeric Pain Rating Scale (NPRS), and Visual Analog Scale (VAS)—were administered at baseline (initial evaluation), Visit 3 (week 3), Visit 6 (week 6), and Visit 7 (week 10).

### 2.5. Intervention

Based on the examination findings, the initial intervention aimed to address upper cervical dysfunction with the following objectives: restoring AA joint mobility, correcting atlas positioning in the transverse plane, improving right AA and lower cervical facet mobility, reducing hypertonicity in the right suboccipital triangle, and decreasing headache intensity. To achieve these goals, manual therapy techniques targeting the upper cervical spine were employed (Table 2).

Muscle energy techniques were employed to facilitate atlas de-rotation by activating specific cervical musculature [18]. The patient was positioned supine with the head in slight extension and left rotation while maintaining the remainder of the cervical spine in a midline position.

In this posture, a 6 s isometric contraction was applied at the left temple to activate the left obliquus capitis inferior. This was immediately followed by a 6 s contraction against resistance at the right temple, intended to activate the right rectus capitis anterior to support continued left atlas rotation. This alternating sequence was repeated six times. Palpation of the suboccipital region was performed during the technique to confirm appropriate muscle engagement. The sequence was then repeated with the head positioned in greater extension and left rotation [18].

Following the de-rotation technique for the atlas, a non-specific supine thrust manipulation was performed targeting the T3–T4 spinal segment. This intervention was selected based on the principle of regional interdependence, as supported by the 2017 Clinical Practice Guidelines for the management of chronic cervicogenic headaches [54,70]. After the thoracic manipulation, the patient received deep friction massage to the suboccipital muscles in the supine position. This was followed by passive tissue lengthening achieved through neck extension combined with suboccipital flexion, resulting in a noticeable reduction in muscle tone.

These initial manual therapy interventions led to a significant improvement in symptoms, with the patient reporting a decrease in headache intensity from 7/10 to 2/10 (Table 1). This supports the hypothesis that upper cervical dysfunction was contributing to her headache presentation [56]. To sustain the benefits of manual therapy, the patient was prescribed an augmented home exercise program. This included active upper cervical rotation to the left in a position of maximal cervical flexion aimed at maintaining AA joint mobility. Additionally, she was instructed to perform supine activation of the deep neck flexor muscles, a task she was already familiar with, using musculoskeletal ultrasound as biofeedback to ensure proper technique. Muscle stretching was utilized to decrease muscular tone starting during visit 3, with a focus on the pectoralis, trapezius, and scalene musculature. Postural training was also incorporated into the treatment plan to enhance her awareness of head, neck, thoracic, and scapular alignment and integration.

Each treatment session lasted approximately 45 min, and the patient completed four sessions over a period of four weeks. By the end of this block, headache frequency had fallen from two to three days per week to one day, and peak intensity was better controlled at 4/10. Every visit began with a reassessment of AA mobility using the FRT and palpation of the atlas transverse processes; these measures served as test-retest indicators of treatment effect. In addition to the previously described upper-cervical techniques, manipulation was applied to address hypomobility of the cervical and upper thoracic segments following the methods of Hartman [71].

A fifth session took place five weeks after the initial visit. At that time, the left AA rotation had improved, and the transverse-plane atlas position appeared more symmetrical. Manual therapy was directed toward residual cervical–thoracic joint restrictions and persistent right-sided myofascial tone. Although upper cervical mechanics had normalized, the patient continued to experience headaches (1 day a week (Table 1)), suggesting a possible non-mechanical contribution. During this visit, she was introduced to external vagus nerve stimulation (eVNS) and its proposed benefits [24,25].

**Figure 2:**
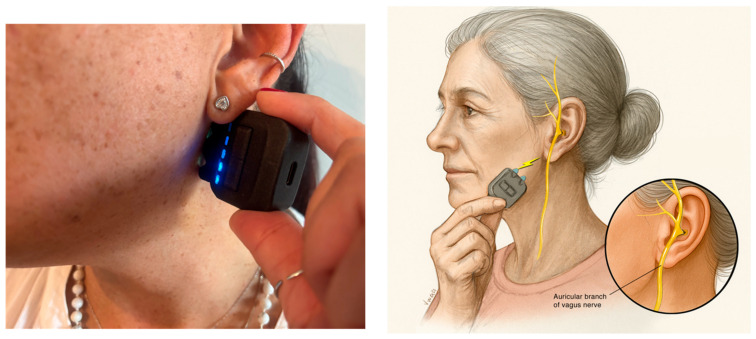
Application of the eVNS directly on the auricular branch of the vagus nerve.

After providing informed consent, she began using a commercially available eVNS device that stimulates the auricular branch of the vagus nerve, producing a mild tingling sensation in the lower ear (Figure 2) [27,28]. The mechanism remains incompletely understood; nevertheless, both neurophysiological and neuromodulatory effects may benefit chronic headaches, and preliminary evidence suggests that eVNS can modulate central pain-processing pathways linked to cervical dysfunction [29]. Because an evidence-based dosage has not been established, she was advised to use the device bilaterally and build up to 10 min of stimulation over four days, applied once on waking and once before sleep, with discretionary daytime use according to the response.

One week later, she reported transient symptom exacerbation after the morning sessions but consistent relief and improved sleep following the evening sessions. Consequently, she restricted use to nightly application, which proved beneficial. Device placement was reviewed bilaterally; no additional manual therapy was required because joint biomechanics remained optimal. Two weeks thereafter, she was headache-free while self-managing with her home exercise program (HEP) and eVNS. One month later, she continued to report no headaches, slept well, and noted markedly reduced anxiety. Cervical and thoracic reevaluation revealed no joint dysfunction. Her HEP was updated with scapular stabilization exercises to address mild winging, and she demonstrated improved postural awareness. She was discharged at that visit. At a one-month follow-up, she remained headache-free, functionally unrestricted, and continued to use the eVNS unit, experiencing a perceived benefit.

## 3. Results

The NDI, HDI, NPRS, and VAS were administered at baseline (initial evaluation), at visit 3 (week 3), visit 6 (week 6), and visit 7 (week 10). Positional assessment of the atlas in the transverse plane, the flexion-rotation test (FRT), active cervical rotation, and deep neck flexor (DNF) strength and endurance were evaluated at each visit. These measures were utilized to guide clinical decision-making and assess treatment effects through repeated testing (Table 1). Clinically meaningful improvements were observed in the NDI, NPRS, and VAS scores.

The NDI improved from 58 to 12 out of 100, indicating a 76.92% reduction in disability. This change exceeded the established minimal clinically important difference (MCID) of 5.5 points [72]. The NPRS for headache intensity decreased from 7 to 0 out of 10, demonstrating a 100% improvement, which also surpassed the MCID threshold of 2.5 points [72]. Likewise, the VAS for self-reported neck pain decreased from 42 to 0 mm (100% improvement), meeting the MCID criterion of 7 mm [72]. Although the MCID for the NDI in individuals with CGH has not been established, the minimal detectable change (MDC) for the HDI in headache populations ranges from 16 to 29 points [47]. In this case, the HDI score improved by 52 points, or 72.22%, thereby exceeding the reported MDC [73,74]. At the initial visit, the patient reported experiencing headaches on two to three days per week. By Visit 3, the frequency had decreased to one day per week, and by Visit 6, she reported no headaches. This headache-free status was maintained through the follow-up at Visit 7, representing a 100% improvement from baseline.

Initially, the atlas appeared right-rotated in the transverse plane, potentially contributing to restricted left AA and cervical rotation. By visits 6 and 7, the atlas had assumed a more symmetrical alignment. AA joint mobility appeared to correlate with atlas position: left AA rotation increased from 33° at baseline to 45° at the final assessment, a 35% improvement. Measuring cervical ROM with a goniometer has been demonstrated to have excellent within-session reliability (ICC2,1 = 0.83 to 0.98) [74]. Right AA rotation remained relatively stable, ranging between 45° and 47°, reflecting a non-significant change of 4%. When combined, total AA rotational movement improved by 18%, though the clinical significance of this combined metric remains unexplored. Future research is warranted to investigate the role of total AA mobility in relation to CGH and other craniovertebral dysfunctions.

Cervical active rotation to the right improved from 67° to 72°, representing a modest 6% gain. Left cervical rotation improved more substantially, from 51° to 70°, a 29% increase that may indicate a clinically meaningful improvement in mobility. This positive change is likely attributable to increased joint motion in the upper cervical region, cervicothoracic junction, and upper thoracic spine (Table 1). However, no cause–effect relationship between the manipulation intervention and the observed mobility gains can be determined using a case report. DNF strength, as assessed by manual muscle testing (MMT), improved from 4/5 to 5/5, representing a 20% gain. While the clinical significance of this change is unclear, the DNF endurance test improved from 14 s to 42 s, representing a 200% increase, which suggests a substantial improvement in neuromuscular control and endurance.

In summary, although causal relationships cannot be established from a single case report, all outcome measures demonstrated improvements from baseline. These changes corresponded with a more neutral atlas position and reductions in both neck pain and headache severity. Further research is recommended to investigate the potential mechanisms underlying these findings, particularly the role of external vagus nerve stimulation (eVNS), pain modulation, and autonomic nervous system regulation in individuals with symptoms.

## 4. Discussion

This case report presents the clinical course and outcomes of a patient with chronic CGH treated with a multimodal approach combining targeted manual therapy and eVNS. The findings demonstrate substantial improvements across pain, function, and neuromuscular control metrics, suggesting potential synergistic benefits of integrating eVNS with mechanical interventions to address both the peripheral and central drivers of CGH.

The pathophysiology of CGH is complex and multifactorial, involving both peripheral musculoskeletal dysfunction and central nervous system sensitization. In this case, notable findings included an initial asymmetry in the transverse position of the atlas and reduced left atlantoaxial (AA) rotation. The positive response to manual techniques aimed at repositioning the atlas and mobilizing the AA joint aligns with previous literature emphasizing the role of upper cervical mobility in modulating CGH symptoms [11,54]. This observation is also consistent with earlier reports linking correction of atlas position to reductions in CGH [18,19]. The observed 35% improvement in left AA rotation, coupled with the normalization of the atlas position in the transverse plane, coincided with significant reductions in neck pain and headache intensity. These findings align with Hall et al.’s diagnostic criteria for CGH, which include pain provocation with cervical motion and restricted AA mobility [8].

Additionally, soft tissue dysfunction, such as increased tone in the suboccipital muscles, may contribute to CGH through myodural bridges, which are fibrous connections linking the suboccipital musculature with the spinal dura mater [13,14]. By influencing dural tension and intracranial mechanosensitivity, suboccipital muscle imbalance may potentiate headache symptoms. Manual therapy targeting these tissues, both via deep friction massage and positional mobilization, likely played a role in reducing mechanical tension and afferent drive to the trigeminocervical complex. A reduction in suboccipital muscle tone has been previously correlated with a reduction of CGH symptoms [75].

Beyond atlas position and mobility, impaired motor control is a hallmark of CGH [76]. The patient in this report demonstrated reduced strength and endurance of the DNF, consistent with patterns observed in individuals with postural syndromes and chronic neck pain. The DNF endurance improved markedly from 14 to 42 s (a 200% increase), suggesting improved neuromuscular function and likely contributing to postural stability and load sharing across the cervical spine. Training of the DNF has been shown to improve sensorimotor control, reduce pain, and enhance cervical proprioception [77,78]. These effects are particularly relevant in CGH, where deficits in joint position sense and altered cervical kinesthesia may perpetuate nociceptive input. The integration of DNF exercises, coupled with posture correction strategies, likely contributed to symptom resolution and improved functional capacity in this patient.

A novel element of this case was the incorporation of eVNS targeting AbV. While manual therapy addressed mechanical dysfunction, the addition of eVNS aimed to modulate autonomic tone and central sensitization. The patient’s improved self-reported sleep, reduced anxiety, and sustained headache relief following eVNS application suggest a beneficial role for autonomic regulation in CGH management. These clinical findings support a 2025 recommendation to consider eVNS as part of the management strategy for individuals with CGH [79]. The vagus nerve plays a central role in maintaining autonomic homeostasis, influencing both cardiovascular and inflammatory pathways [24]. Through stimulation of the AbV, eVNS activates afferents projecting to the nucleus tractus solitarius (NTS), modulating activity in the locus coeruleus and periaqueductal gray structures involved in descending pain inhibition [29].

Our findings in this case are consistent with recent clinical studies supporting the efficacy of eVNS in treating headache disorders. Moderate-to-high-quality evidence indicates that eVNS can reduce headache frequency and severity while maintaining a favorable side-effect profile [27,29]. Notably, Cornelison et al. [34] reported that noninvasive vagus stimulation reduced trigeminal pain signaling with similar effectiveness to morphine, highlighting the potential of eVNS as a non-pharmacologic pain management strategy. In this case, the patient initially reported transient discomfort with morning stimulation but experienced sustained symptom relief with nighttime use. This response highlights the importance of individualized titration and monitoring in eVNS application, supporting emerging recommendations for patient-centered dosing strategies. Future studies should examine the benefit of unilateral versus bilateral vagus nerve stimulation, as well as the duration of stimulation on each side, which influences treatment outcomes and helps determine optimal parameters.

This case illustrates the potential value of a multimodal approach that combines manual therapy to address mechanical contributors and eVNS to influence central sensitization and autonomic dysfunction. This integrative framework reflects contemporary models of headache management, which recognize the multidimensional nature of pain, encompassing nociceptive, neuropathic, and affective components [21]. The patient reported improved sleep, reduced anxiety, and enhanced tolerance to postural demands, effects consistent with parasympathetic activation via auricular vagus stimulation. While manual therapy provided immediate symptom relief, the sustained effects observed with eVNS suggest that targeting neurophysiological mechanisms may enhance long-term outcomes. Additionally, the patient’s improvement in postural control and self-efficacy likely reflects an interaction between reduced pain perception and improved neuromotor control. Such integrative models have been advocated in recent literature. Straube and Eren [37] highlight the role of transcutaneous vagus stimulation in reducing chronic headache and musculoskeletal pain by modulating autonomic balance and cortical excitability. Similarly, Austelle et al. [25] emphasize the future potential of vagus stimulation in addressing refractory pain conditions via targeted neuroimmune modulation.

### Limitations

As a single-case report, this study cannot establish causality or generalize findings to broader populations. While improvements were observed across multiple domains, it is unclear which intervention contributed most significantly to symptom resolution. Additionally, placebo effects, natural recovery, and regression to the mean cannot be ruled out as possible explanations for the observed outcomes. Another limitation lies in the absence of objective autonomic metrics, such as heart rate variability or pupillometry, to quantify the physiological impact of eVNS. Future studies incorporating such measures would help clarify the mechanisms through which the vagus nerve stimulation influences CGH and neck pain. Additionally, the optimal frequency, duration, and stimulation parameters for eVNS have yet to be established and should be investigated in randomized controlled trials. This case also did not employ psychometric scales to objectively assess variables such as anxiety and sleep quality; inclusion of these measures in future research would provide a more comprehensive understanding of treatment effects.

Despite these limitations, this case contributes to the growing body of literature supporting the use of neuromodulation in managing complex headache disorders. It also underscores the importance of integrating patient-reported outcomes with biomechanical and neurophysiological assessments to guide individualized care. Future studies incorporating larger sample sizes, physiological monitoring, and standardized dosing protocols are needed to validate and expand upon these preliminary findings.

## 5. Conclusions

This case report illustrates the potential benefits of integrating external eVNS into a physiotherapy-led multimodal approach for chronic CGH. While manual therapy, postural retraining, and targeted exercise addressed upper cervical dysfunction and neuromuscular deficits, the addition of eVNS likely modulated autonomic tone and enhanced symptom resolution. Clinically meaningful improvements were observed in pain, disability, and motor control, with sustained benefits at follow-up.

These findings support a biopsychosocial framework for the management of CGH, emphasizing the interplay between biomechanical impairments and neurophysiological regulation. Although causality cannot be established, the temporal association and magnitude of the observed changes suggest that eVNS may serve as a valuable adjunct for patients with persistent CGH who do not respond to conventional therapy. Further research is warranted to explore the optimal dosing parameters, mechanisms of action, and long-term effects of eVNS combined with manual therapy and therapeutic exercise.

### Patient Perspective

At the conclusion of the treatment, the patient expressed high satisfaction with her clinical progress and personalized care. She reported that the integration of hands-on therapy with targeted home exercises helped her better understand the musculoskeletal contributors to her headaches. The use of eVNS was described as “easy to integrate” into her daily routine and “surprisingly calming,” particularly during nighttime use. She expressed relief in regaining control over her symptoms and appreciated the physiotherapy-led explanation of the autonomic mechanisms involved. The patient described the overall experience as “empowering” and stated that she felt “much more confident” in managing future symptoms independently.

## Figures and Tables

**Table 1:** Examination data, percentage of change, and intervention data. HDI—Headache disability inventory, NDI—Neck Disability Index, VAS—Visual Analogue Scale, DNF—deep neck flexor, rot—rotation, AA—atlantoaxial, MMT—manual muscle testing.

Visits	1	2	3 Reexamine	4	5	6 Reexamine After 2 Weeks	7 Reexamine 1 Month Later	% Change
Assessments								
NDI	58/100	X	32/100	X	X	18/100	12/100	76.92%
HDI	72/100	X	52/100	X	X	22/100	20/100	72.22%
Headache (0–10 scale)	7/10	5/10	4/10	4/10	2/10	0/10	0/10	100%
Headache days	2–3 days/week	X	1 day a week	X	X	0 days/week	0 days/week	100%
Neck pain (VAS)	42 mm	26 mm	25 mm	16 mm	10 mm	4 mm	0 mm	100%
Palpation for position atlas	(+) right rot	(+) right rot	(−) right rot	(+minimal) right rot	(+minimal) right rot	Symmetrical	Symmetrical	
Cervical rot right	67	72	70	72	73	71	72	6%
Cervical rot left	51	55	52	62	65	72	70	29%
AA rot right	43	45	43	44	45	45	47	4%
AA rot left	29	33	38	38	42	44	45	35%
Total AA rot	72	78	81	82	87	89	92	18%
DNF MMT	4/5	4/5	4+/5	4+/5	4+/5	5/5	5/5	20%
DNF endurance	14 s	18 s	21 s	22 s	30 s	39 s	42 s	300%

**Table 2:** Intervention data. AA—atlantoaxial, AO—atlantooccipital, Evns—external valgus nerve stimulation, Manip—manipulation, DNF—Deep neck flexor, HEP—Home exercise program, manip—manipulation.

Interventions						
1	2	3 Reexamine	4	5	6 Reexamine After 2 Weeks	7 Reexamine 1 Month Later
-AA/AO muscle energy -Distraction manip AA, C5-C7 -T3-4 R manip -Myofascial sub occ	-AA/AO muscle energy -Distraction manip C5-C7 -T3-4 R manip -Myofascial sub occ	-AA/AO muscle energy -Distraction C5-C7 -T3-4 R manip -Myofascial sub occ	-Distraction manip C5-C7 -T3-4 R manip -Myofascial cervical spine and soft tissue -stretching supine subocc, trapezius and levator. -Standing pec stretching and posture control. -eVNS instruction and application	-Review of HEP, including self-mobilization AA -Posture correction -eVNS instruction and application	eVNS instruction and application	eVNS instruction and application
Same as visit 1	-Pec stretch 3 × 30 90/140 degrees. -Trap stretch 3 × 30 -Scaleni stretch 3 × 30 -Middle and rhomboid strength with green theraband. -Pushup through for serratus. -General standing posture	-Pec stretch 3 × 30 90/140 degrees. -Trap stretch 3 × 30 -Scaleni stretch 3 × 30 -Middle and rhomboid strength with green theraband. -DNF supine -PNF head -Scapular stabilization. -Standing/sitting posture	-Pec stretch 3 × 30 90/140 degrees. -Trap stretch 3 × 30 -Scaleni stretch 3 × 30 -Middle and rhomboid strength with green theraband. -Continue with cervical stabilization ex -Scapular stabilization. -Standing/sitting posture	-Continue previous exercise program with focus on upright posture	-Continue previous exercise program with focus on upright posture	

## Data Availability

All data generated or analyzed during this case are included in this published article.

## Abbreviations

The following abbreviations are used in this manuscript:

CGH	Cervicogenic Headache
eVNS	external Vagus Nerve Stimulation
AA	Atlantoaxial
ANS	Autonomic Nervous System
SNS	Sympathetic Nervous System
VNS	Vagus Nerve Stimulation
AbV	Auricular branch vagus
NPRS	Numeric Pain Rating Scale
VAS	Visual Analogue Scale
NDI	Neck Disability Index
HDI	Headache Disability Index
AROM	Active Range Of motion
FRT	Flexion Rotation Test
HEP	Home Exercise Program
DNF	Deep Neck Flexors
MCID	Minimal Clinically Important Difference
